# Family Caregiver Assessments: What Have We Learned From Assessments Across Populations and Health Conditions?

**DOI:** 10.1093/geroni/igaa057.2410

**Published:** 2020-12-16

**Authors:** Gabriela Prudencio, Laura Gitlin

## Abstract

This symposium will bring together research on assessments of family caregivers for older individuals with different health conditions and discuss the components of effective assessments. Comprehensive characterizations of caregivers are essential due to an increase in the demand on caregivers, and how intense care contexts contribute to the caregiver’s decline in health and diminished capacity to provide quality care. According to Caregiving in the U.S. 2020 (n=1,400), only 13% of caregivers were asked by healthcare professionals what they needed to take care of themselves. Peer-reviewed studies have reported that caregivers are often reluctant to self-identify and to ask for the help that they need for themselves and those in their care. Since supports to caregivers have historically relied on this self-identification, the first presentation (Grace Whiting) will focus on the work NAC has done to build pathways between caregivers and supportive services to increase availability, accessibility, and patient-centeredness. The second presentation (Esther Friedman) will identify and discuss the barriers to fully incorporating family caregivers into the health care team, as well as the solutions for removing barriers. The next two presentations, respectively, will focus on characterizing the prevalence, burden, and unmet needs of caregivers of cancer patients (Erin Kent), and the unmet needs of families of adults with intellectual and development disabilities (Tamar Heller). The final presentation will explore caregiver readiness in dementia care using the Tailored Activity Program (TAP) and how TAP interventionists can use readiness scores to determine caregiver’s capacity (Katherine Marx).

